# Cellular Levels and Binding of c-di-GMP Control Subcellular Localization and Activity of the *Vibrio cholerae* Transcriptional Regulator VpsT

**DOI:** 10.1371/journal.ppat.1002719

**Published:** 2012-05-24

**Authors:** Nicholas J. Shikuma, Jiunn C. N. Fong, Fitnat H. Yildiz

**Affiliations:** Department of Microbiology and Environmental Toxicology, University of California, Santa Cruz, Santa Cruz, California, United States of America; Stanford University School of Medicine, United States of America

## Abstract

The second messenger, cyclic diguanylate (c-di-GMP), regulates diverse cellular processes in bacteria. C-di-GMP is produced by diguanylate cyclases (DGCs), degraded by phosphodiesterases (PDEs), and receptors couple c-di-GMP production to cellular responses. In many bacteria, including *Vibrio cholerae*, multiple DGCs and PDEs contribute to c-di-GMP signaling, and it is currently unclear whether the compartmentalization of c-di-GMP signaling components is required to mediate c-di-GMP signal transduction. In this study we show that the transcriptional regulator, VpsT, requires c-di-GMP binding for subcellular localization and activity. Only the additive deletion of five DGCs markedly decreases the localization of VpsT, while single deletions of each DGC do not impact VpsT localization. Moreover, mutations in residues required for c-di-GMP binding, c-di-GMP-stabilized dimerization and DNA binding of VpsT abrogate wild type localization and activity. VpsT does not co-localize or interact with DGCs suggesting that c-di-GMP from these DGCs diffuses to VpsT, supporting a model in which c-di-GMP acts at a distance. Furthermore, VpsT localization in a heterologous host, *Escherichia coli*, requires a catalytically active DGC and is enhanced by the presence of VpsT-target sequences. Our data show that c-di-GMP signaling can be executed through an additive cellular c-di-GMP level from multiple DGCs affecting the localization and activity of a c-di-GMP receptor and furthers our understanding of the mechanisms of second messenger signaling.

## Author Summary

C-di-GMP is a ubiquitous intracellular signaling molecule in bacteria and controls diverse cellular processes including biofilm formation, motility and virulence. The genomes of many bacteria often contain numerous genes encoding proteins predicted to produce and degrade c-di-GMP. However, it is currently unclear how a bacterial cell orchestrates multiple c-di-GMP signaling proteins to elicit a specific cellular response. The microbial pathogen, *Vibrio cholerae*, contains a multitude of c-di-GMP proteins and c-di-GMP signaling is likely important for the bacterium to cause the deadly diarrheal disease called cholera. In the present study, we define the requirements for c-di-GMP signal transduction in *V. cholerae*. We identify specific c-di-GMP proteins that additively stimulate the subcellular localization and activity of the c-di-GMP binding protein and transcriptional regulator, VpsT. We further show that c-di-GMP signaling does not require the interaction of c-di-GMP signaling components. However, a common cellular level of c-di-GMP contributes to VpsT localization and activity. This is the first account of the subcellular localization of a transcriptional regulator modulated by c-di-GMP binding. Furthermore, this study establishes that c-di-GMP signal transduction can act at a distance through a common cellular level of c-di-GMP and defines how an intracellular second messenger can regulate cellular processes in bacteria.

## Introduction

Second messengers are small diffusible signaling molecules that are produced or degraded in response to external stimuli and relay information to a receptor to elicit a specific cellular response [1]. The cyclic nucleotide cyclic diguanylate (c-di-GMP) is a bacterial second messenger that controls the transition between a free living and biofilm lifestyle [2], [3]. C-di-GMP is produced by diguanylate cyclases (DGCs), containing GGDEF domains, and degraded by phosphodiesterases (PDEs), containing EAL or HD-GYP domains. Cellular c-di-GMP is sensed by receptors that interact with downstream targets to affect cellular functions. C-di-GMP signaling often involves numerous GGDEF, EAL or HD-GYP domain containing proteins and receptors [4], and previous reports suggest that the compartmentalization of c-di-GMP signaling components could facilitate the activation of specific cellular processes [3], [5], [6]. However, it is currently unclear whether compartmentalization is required to mediate c-di-GMP signal transduction in bacteria.

Recent advances in the identification of c-di-GMP receptors have helped define the mechanisms by which c-di-GMP mediates downstream processes. These receptors include riboswitches [7] and proteins that contain various binding domains. PilZ domains are known to bind c-di-GMP and proteins harboring these domains modulate cellular processes such as motility through protein-protein interactions with the flagellar motor complex [8]–[10]. Proteins containing degenerate GGDEF or EAL domains, which have lost their enzymatic activity, are also known to be c-di-GMP receptor proteins. In *Pseudomonas fluorescens*, LapD binds c-di-GMP through a degenerate EAL domain and modulates the cell surface association of an adhesin through direct interactions with a periplasmic protease [11]–[13]. The degenerate GGDEF domain containing protein CdgG was shown to regulate biofilm formation in *Vibrio cholerae* [14]. C-di-GMP can also regulate gene expression by binding transcriptional regulators such as the Crp homolog Clp [15] or FleQ [16]. Although the identities of many c-di-GMP receptor proteins are known, the mechanisms of c-di-GMP-mediated signal transduction and gene regulation are not fully understood.

In *V. cholerae*, the bacterial pathogen that causes the disease cholera, c-di-GMP regulates biofilm formation, motility and virulence [17]–[19]. The *V. cholerae* genome contains 31 genes encoding proteins with GGDEF domains, 11 genes encoding proteins with EAL domains, 10 genes encoding proteins with both GGDEF and EAL domains and 9 genes encoding proteins with HD-GYP domains [14], [20]. Recently, we characterized VpsT, which is a key c-di-GMP receptor known to regulate biofilm formation in *V. cholerae* [21]. Biofilm formation in *V. cholerae* requires the biosynthesis of *Vibrio* polysaccharide (VPS) [22], [23], and VpsT activates *vps* expression through direct binding of the *vpsL* promoter [21], [24]. VpsT is a novel member of the FixJ, LuxR and CsgD family of transcriptional regulators that contains a c-di-GMP binding motif and a 6^th^ alpha helix at its N-terminal receiver domain that stabilizes homodimerization [21]. These features make VpsT unique compared to other response regulators and c-di-GMP binding proteins.

In this study, we report that VpsT requires c-di-GMP binding and subcellular localization to regulate gene expression. The wild-type VpsT localization pattern is dependent on c-di-GMP binding, c-di-GMP-stabilized dimerization, and the VpsT DNA binding domain. We also show that VpsT does not co-localize or interact with DGCs. Instead, multiple DGCs contribute additively to a cellular c-di-GMP concentration, which affects the localization and activity of the c-di-GMP receptor protein, VpsT.

## Results/Discussion

### VpsT Is Subcellularly Localized and Multiple DGCs Contribute Additively to VpsT Localization

**Figure 1:**
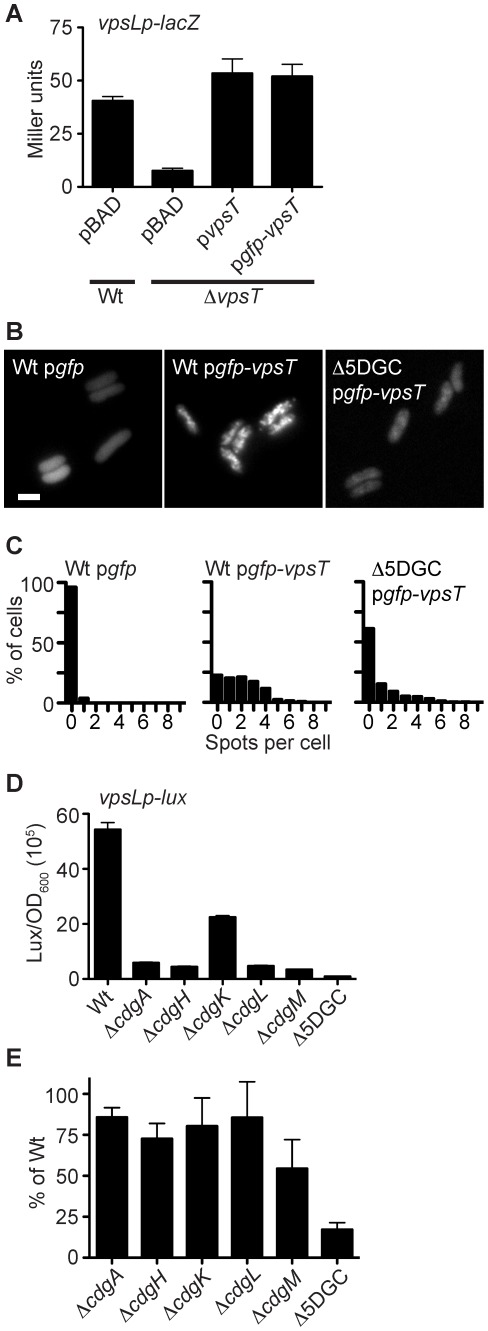
VpsT Localization is Dependent on Multiple Diguanylate Cyclases. (A) The expression of a chromosomal *vpsL* promoter-*lacZ* fusion was measured in wild type (Wt) or Δ*vpsT* strains containing pBAD vector alone, or pBAD containing *vpsT* or *gfp*-*vpsT* using β-galactosidase assays. One representative experiment of three biological replicates is shown. Error bars indicate standard deviations of eight technical replicates. (B) Representative epifluorescence micrographs are shown of the subcellular localization of GFP or GFP-VpsT fusion protein in wild-type or Δ5DGC *V. cholerae* strains. Marker is 2 µm. (C) Single-cell quantification of subcellular fluorescence localization. The number of spots per cell is shown as a histogram for wild type or Δ5DGC strains expressing GFP or GFP-VpsT. Data are acquired from at least 3 independent experiments and quantification was performed on at least 150 cells per treatment. (D) Expression of the *vpsL* promoter fused to a *lux* reporter operon in wild-type *V. cholerae* (Wt) or strains containing single in-frame deletions of the genes encoding DGCs *cdgA*, *cdgH*, *cdgK*, *cdgL*, *cdgM* or a strain containing in-frame deletions all 5 DGCs (Δ5DGC). Expression is reported in luminescence counts min^−1^ ml^−1^/OD_600 nm_. One representative experiment of three biological replicates is shown. Error bars indicate standard deviations of four technical replicates. (E) Percent c-di-GMP levels of single in-frame deletion DGC mutants or the Δ5DGC strain compared to wild type *V. cholerae* using high-performance liquid chromatography-tandem mass spectrometry. Error bars indicate standard deviations of three biological replicates.

We hypothesized that the c-di-GMP receptor protein, VpsT, is subcellularly localized, and this localization facilitates c-di-GMP signal transduction. To determine whether VpsT is subcellularly localized, we constructed an N-terminal tagged green fluorescent protein (GFP)-VpsT fusion. Expression of *gfp*-*vpsT* recovered *vpsL* expression in a Δ*vpsT* strain (Figure 1A). *vpsL* is the first gene in the *vps-*II operon and VpsT directly binds to the upstream regulatory region of this gene [21], [22]. Expression of *vpsL* was similar between strains expressing *gfp-vpsT* or *vpsT* alone indicating that our fusion protein is functional. When observed by fluorescence microscopy, GFP-VpsT formed a pattern of localization within the cell (Figure 1B), while a strain expressing GFP exhibited homogenous fluorescence throughout the cytoplasm. We confirmed that this localization was not due to different cellular protein concentrations, as levels of GFP-VpsT were similar to levels of GFP alone (Figure S1). A census of more than 150 cells per treatment showed that cells expressing GFP-VpsT contained more spots per cell when compared to cells expressing GFP alone when quantified using MicrobeTracker software (Figure 1C) [25]. GFP-VpsT localization also exhibited a higher ratio of maximum to average fluorescence intensity across the length of individual cells when compared to cells expressing GFP alone (Figure S1). These results indicate that GFP-VpsT is subcellularly localized.

The striking number of GGDEF, EAL and HD-GYP domain containing proteins present in many bacteria is thought to generate flexibility in signal transduction, allowing multiple sensory inputs to be fed through a single diffusible signaling molecule [4]. Since VpsT is a c-di-GMP binding protein and is subcellularly localized, we wondered whether specific DGCs or PDEs are important for this localization pattern. We therefore measured expression of the *vpsL* promoter in wild-type *V. cholerae* and 52 strains containing in-frame deletions of each gene in the *V. cholerae* genome encoding proteins with GGDEF, EAL or GGDEF and EAL domains. Of the strains examined, 5 DGC deletion strains showed a 2-fold or greater decrease in expression of *vpsL* (Figure 1D and S2), namely the previously characterized genes encoding DGCs *cdgA* (VCA0074), *cdgH* (VC1067), *cdgK* (VC1104) and *cdgL* (VC2285) [14], [26], [27], and a predicted DGC, VC1376, which we name here, *cdgM*. Furthermore, c-di-GMP levels decreased between 86% and 54% in each single DGC deletion strain when compared to wild type (Figure 1E). These results show that multiple DGCs are involved in *vps* regulation and thus identified likely candidate DGCs important for VpsT localization.

We then observed VpsT localization in strains lacking each of the 5 DGCs important for *vpsL* expression. VpsT localization was not markedly altered in any strain containing a single DGC deletion (Figure S1). We then reasoned that VpsT localization may not be dependent on a single DGC, but instead, multiple DGCs contribute additively to VpsT localization. Therefore, we created a strain where all 5 DGCs are deleted in combination, designated Δ5DGC. Δ5DGC exhibited a lower *vpsL* expression than any single DGC mutant strain (Figure 1D). Moreover, c-di-GMP levels were significantly decreased (17%) in the Δ5DGC strain compared to wild type (Figure 1E). In the Δ5DGC strain, GFP-VpsT localization was reduced and the number of spots per cell and ratio of maximum to average fluorescence intensity were markedly lower compared to wild type expressing the same fluorescent fusion protein (Figure 1B, 1C and S1). This change in GFP-VpsT localization was not due to different cellular protein concentrations, as GFP-VpsT levels were similar to levels of GFP alone in the Δ5DGC strain (Figure S1). These results indicate that no single DGC is sufficient to cause VpsT mis-localization, and instead, multiple DGCs additively impact the GFP-VpsT localization pattern. The number of spots per cell in the Δ5DGC strain was not completely diminished, and we speculate that a low level of c-di-GMP is still present in the cell due to remaining DGCs, which facilitate VpsT localization. Alternatively, a range of VpsT target promoters that differ in their affinities for the active regulator could cause this localization pattern. Above observations of VpsT localization and activity suggest that VpsT function is dependent on reaching a critical cellular c-di-GMP threshold. Thus we wondered whether a single DGC could rescue *vpsL* expression in the Δ5DGC strain. When *cdgA* was expressed on a plasmid in the Δ5DGC mutant, *vpsL* expression was recovered in the Δ5DGC strain when compared to the Δ5DGC mutant harboring the vector alone (Figure S3). These results suggest that one DGC can rescue a cellular level of c-di-GMP for the activation of *vpsL* expression in the Δ5DGC strain.

In our survey of DGC and PDE mutants, we also observed multiple PDEs to be negative regulators of *vps* expression (Figure S2), consistent with previous work [26], [28]–[30]. However, strains harboring deletions of three of these genes encoding PDEs, *mbaA*, *rocS* and *cdgC* individually or in combination, exhibited no significant alteration in GFP-VpsT localization pattern (Figure S4). Therefore, an upper c-di-GMP concentration limit may exist, after which, further VpsT localization is not observable. Alternatively, the experimental system might be saturated, and no further localization can be observed.

VpsT as a response regulator is not unique in its capacity to subcellularly localize in response to specific stimuli or modification. CsgD from *Salmonella enterica* was shown to form foci associated with the membrane in a subpopulation of cells in response to cell aging [31]. WspR from *Pseudomonas aeruginosa* was shown to localize to foci in response to phosphorylation [32]. OmpR from *Escherichia coli* subcellularly localizes in response to the presence and activity of its cognate histidine kinase, EnvZ [33]. Whereas typical response regulators, such as OmpR, are activated by a single major cognate histidine kinase [34], VpsT localization and activity is modulated in response to c-di-GMP produced by multiple DGCs. These results are consistent in the context of second messenger signaling, where multiple independent inputs can be fed through a single diffusible signaling molecule to elicit a specific cellular response [1].

### VpsT, CdgA and CdgH Do Not Form a Complex

**Figure 2:**
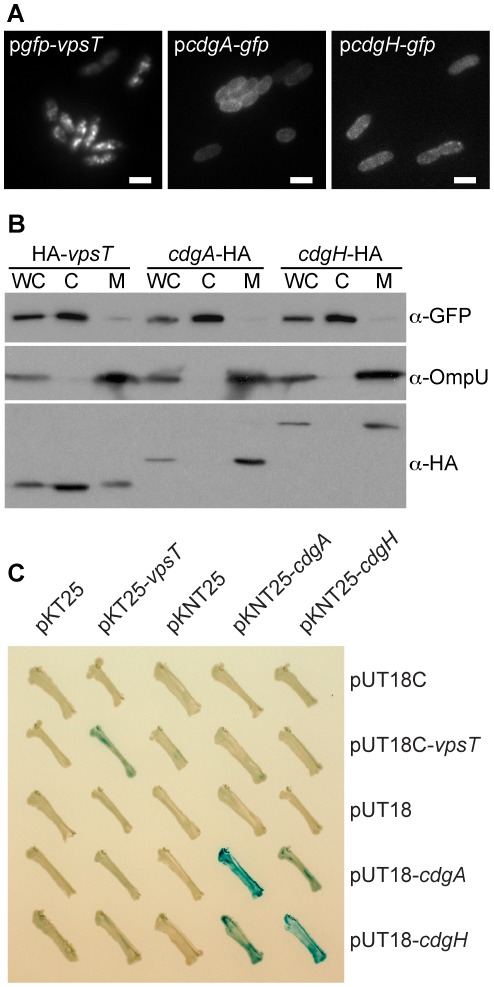
VpsT Does Not Interact with CdgA or CdgH Directly. (A) Representative epifluorescence micrographs of wild-type *V. cholerae* expressing GFP-VpsT, CdgA-GFP or CdgH-GFP fusion proteins. Marker is 2 µm. (B) Subcellular fractionation of *V. cholerae* strains containing *vpsT*, *cdgA* or *cdgH* tagged with an HA epitope in their native chromosomal loci. Western immunoblot was performed on cellular fractions representing whole cell (WC), cytoplasmic (C) and total membrane (M) fractions. HA-tagged proteins were detected using a polyclonal anti-HA antibody. *gfp* was constitutively expressed from a chromosomal locus. GFP was detected using monoclonal anti-GFP antibody and is used as a cytoplasmic fraction control. OmpU was detected using a polyclonal anti-OmpU antibody and is used as a total membrane fraction control. One representative experiment of three biological replicates is shown. (C) Bacterial two-hybrid analysis of VpsT, CdgA and CdgH. Reconstitution of CyaA, indicative of protein-protein interaction, was detected by β-galactosidase activity on LB plates containing ampicillin (100 µg/ml), kanamycin (50 µg/ml), IPTG (500 µM) and X-gal (40 µg/ml). Plates were incubated at 30°C for 48 h.

It is proposed that the subcellular compartmentalization of c-di-GMP signaling components might allow c-di-GMP to act locally on specific cellular processes such as motility or biofilm formation [5], [35]. C-di-GMP signaling proteins could exert their effects by participating in complexes that include signal producers (DGC), removers (PDE), receptors, and/or targets [3], [6]. To determine if co-localization occurs between DGCs activating VpsT and the c-di-GMP receptor, VpsT, we analyzed their subcellular localization. We chose CdgA and CdgH, two DGCs that affect *vps* expression (Figure 1D) and have demonstrated DGC activity (Shikuma and Yildiz, unpublished data) [14]. To observe the subcellular localization of CdgA and CdgH we constructed C-terminal tagged CdgA-GFP and CdgH-GFP fusions. Both *cdgA-gfp* and *cdgH-gfp* were able to complement in-frame deletions of *cdgA* and *cdgH*, respectively (Figure S5), indicating that our fusion proteins are functional. When observed by fluorescence microscopy, CdgA-GFP and CdgH-GFP both appeared to localize to the cell membrane (Figure 2A). Consistent with these results, both CdgA and CdgH are predicted to contain 2 and 1 transmembrane domains, respectively [36].

To corroborate these results, we performed cellular fractionation and western blot analysis to identify the subcellular location of VpsT, CdgA and CdgH. We therefore created strains containing an N-terminal HA tagged *vpsT*, a C-terminal HA tagged *cdgA* or a C-terminal HA tagged *cdgH* in their native chromosomal loci. Strains containing each fusion protein exhibited similar *vpsL* expression when compared to wild type (Figure S5). Both CdgA-HA and CdgH-HA localized to the total membrane fraction, as predicted (Figure 2B). In contrast, HA-VpsT localized mostly to the cytoplasmic fraction, but a lower level also consistently appeared in the total membrane fraction. To determine whether VpsT localization is dependent on the presence of specific DGCs or c-di-GMP levels, we performed a cellular fractionation of wild-type and Δ5DGC strains and probed for HA-VpsT levels. HA-VpsT localization was not different between wild-type and Δ5DGC strains (Figure S6), suggesting that the 5 DGCs or c-di-GMP levels are not important for the localization of VpsT to specific cellular fractions.

Although VpsT resides mainly in a different subcellular region of the cell when compared to CdgA or CdgH, it is possible that transient interactions between these proteins contribute to specificity in c-di-GMP signaling. To address whether VpsT can interact with CdgA or CdgH directly, we performed a bacterial two-hybrid analysis using a system suited to identify protein-protein interactions, even under the condition where one or both proteins are membrane bound [37]. Using bacterial two-hybrid vectors, VpsT, CdgA and CdgH were fused to the T18 or T25 complementary fragments of *Bordetella pertussis* adenylate cyclase (CyaA). Interaction between co-expressed proteins of interest in *E. coli* reconstitute a functional CyaA, leading to cAMP production [38]. As expected, a signal indicative of interaction of VpsT with itself was observed by colorimetric blue production on LB agar containing bromo-chloro-indolyl-galactopyranoside (X-gal), as well as quantitatively using β-galactosidase assays (Figure 2C and S7). Interaction of CdgA with itself and CdgH with itself was also observed (Figure 2C and S7). These results were expected as DGCs from other bacteria were shown previously to catalyze c-di-GMP production as dimers [39], [40]. Interestingly, *E. coli* containing CdgA and CdgH on complementary plasmids exhibited increased β-galactosidase production, suggesting that these DGCs might interact, however the physiological relevance of this observation is unclear at this point. Importantly, strains expressing both VpsT and CdgA or VpsT and CdgH did not exhibit increased cAMP production, even when the reciprocal exchange of fusion domains was performed (Figure 2C and S7). These results suggest that VpsT does not interact directly with CdgA or CdgH.

### VpsT Requires c-di-GMP Binding for Subcellular Localization and Activity

**Figure 3:**
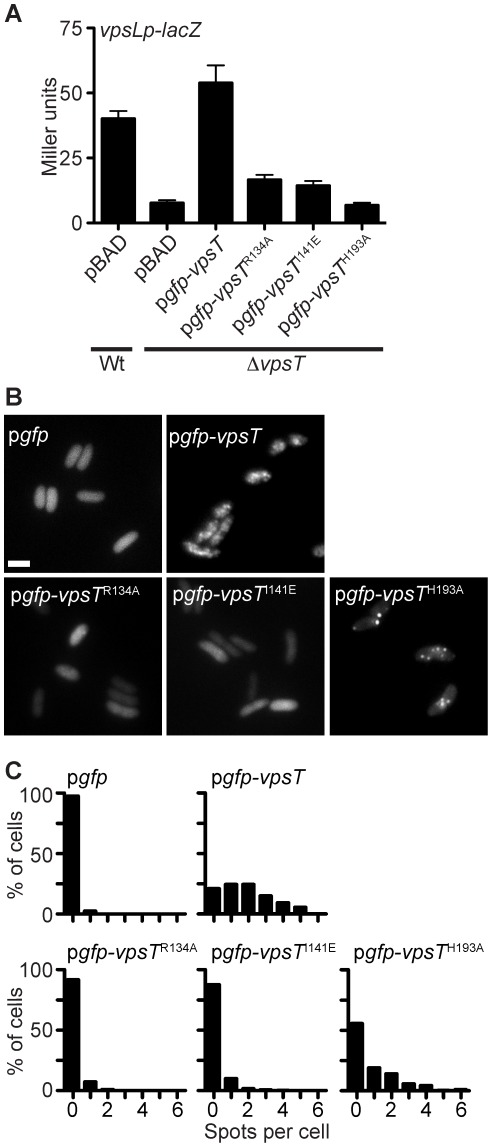
The Subcellular Localization of VpsT is Dependent on c-di-GMP Binding and DNA Binding Residues. (A) The expression of a chromosomal *vpsL* promoter-*lacZ* fusion was measured in wild type (Wt) or Δ*vpsT* strains containing pBAD vector alone, or pBAD containing *gfp* fused to wild type and mutated versions of *vpsT* using β-galactosidase assays. A R134A mutation disrupts c-di-GMP binding and an I141E mutation abolishes c-di-GMP-dependent dimerization. H193A lies in the DNA binding domain of VpsT. (B) Subcellular localization of GFP, GFP-VpsT or GFP-VpsT containing the indicated point mutations, expressed in *V. cholerae* Δ*vpsT*. Representative epifluorescence micrographs are shown. Marker is 2 µm. (C) Single-cell quantification of subcellular fluorescence localization. The number of spots per cell is shown as a histogram for Δ*vpsT* strains expressing GFP, GFP-VpsT or GFP-VpsT containing the indicated point mutations. Data are acquired from at least 3 independent experiments and quantification was performed on at least 150 cells per treatment.

We next wondered whether VpsT localization is dependent on specific domains and/or interfaces important for VpsT function. Mutations in residues required for c-di-GMP binding (VpsT^R134A^) or c-di-GMP-stabilized dimerization (VpsT^I141E^) were unable to complement a Δ*vpsT* mutation (Figure 3A), consistent with our previous findings [21]. When observed by fluorescence microscopy, both GFP-VpsT^R134A^ and GFP-VpsT^I141E^ mutants exhibited a homogenous fluorescence throughout the cytoplasm, possessed almost no spots per cell, and showed a significantly lower maximum to average fluorescence intensity ratio when compared to strains expressing a wild-type GFP-VpsT fusion (Figure 3B, 3C and S8). VpsT contains a C-terminal helix-turn-helix (HTH) DNA binding domain and H193 of VpsT aligned with other histidine residues in the LuxR/FixJ superfamily shown previously to be required for DNA binding (Figure S8) [41], [42]. A strain harboring GFP-VpsT^H193A^ was unable to induce *vps* expression (Figure 3A) and appeared to localize to foci that were more dispersed throughout the cell when compared to wild type GFP-VpsT (Figure 3B). The number of spots per cell and the ratio of maximum to average fluorescence intensity of the GFP-VpsT^H193A^ expressing strain were decreased compared to wild-type GFP-VpsT (Figure 3C and S8). Therefore, VpsT localization, albeit different than that of the wild-type localization pattern, can still occur in the absence of DNA binding. The subcellular localization patterns were not due to differential protein levels, as cellular concentrations of wild-type GFP-VpsT were similar to GFP-VpsT with R134A, I141E or H193A point mutations (Figure S8). Taken together, our results indicate that the wild-type VpsT localization pattern is dependent on c-di-GMP binding and DNA binding. These results suggest that VpsT forms oligomers on DNA binding sites distributed on the *V. cholerae* chromosomes and the localization pattern is due to binding of VpsT to its target sequences on the genome.

### VpsT Localization in a Heterologous Host Depends on Cellular c-di-GMP Levels

**Figure 4:**
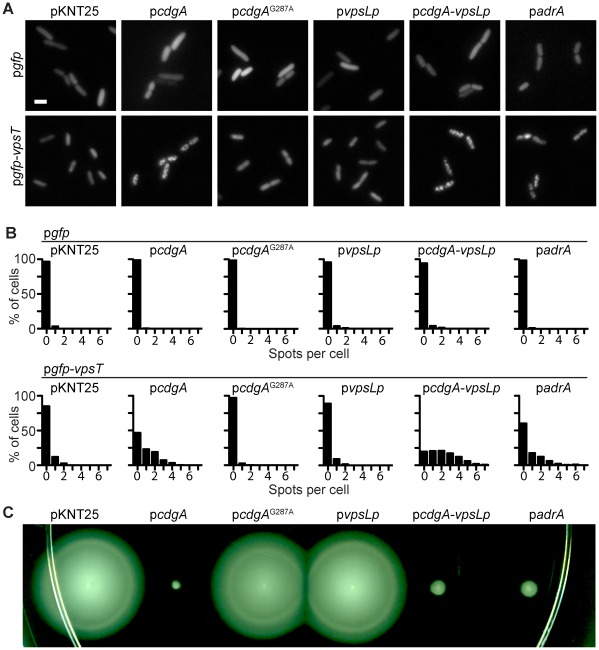
VpsT Localization in a Heterologous Host Depends on c-di-GMP. (A) Representative epifluorescence micrographs of *E. coli* strains expressing GFP or GFP-VpsT and containing a pKNT25 compatible plasmid or pKNT25 harboring *cdgA*, *cdgA* ^G287A^, *vpsL* promoter (*vpsLp*), *cdgA* and *vpsLp* or *adrA* from *S. typhimurium*. Marker is 2 µm. (B) Single-cell quantification of subcellular fluorescence localization. The number of spots per cell is shown as a histogram for *E. coli* strains containing the indicated plasmids. Data are acquired from at least 3 independent experiments and quantification was performed on at least 150 cells per treatment. (C) Representative motility phenotypes of *E. coli* expressing the indicated plasmids grown on soft agar plates containing kanamycin and 10 µM IPTG at 37°C for 12 h.

To determine whether there are other factors responsible for VpsT localization in *V. cholerae*, we expressed GFP-VpsT in *E. coli*. GFP-VpsT was surprisingly homogenous throughout the cytoplasm when expressed in *E. coli* in contrast to the same construct expressed in *V. cholerae* (data not shown), suggesting that the localization of VpsT requires cellular components or a cellular environment provided by the *V. cholerae* cell. We then hypothesized that the localization of VpsT might either require increased levels of c-di-GMP or specifically require a DGC important for biofilm formation in *V. cholerae*. A compatible plasmid that expresses *cdgA* from an IPTG inducible promoter was therefore introduced into *E. coli* containing GFP-VpsT. Strains expressing CdgA showed a marked decrease in motility when compared to strains containing vector alone (Figure 4C), indicating that CdgA is functional as a DGC in *E. coli*. When observed by fluorescence microscopy, GFP-VpsT formed foci in the presence of CdgA in *E. coli* (Figure 4A). This strain exhibited an increase in the number of spots per cell and a significantly increased ratio of maximum to average fluorescence intensity compared to a strain with GFP-VpsT and an empty compatible plasmid (Figure 4A, 4B and S9). To determine whether VpsT localization is dependent on the catalytic activity of CdgA, we also expressed CdgA containing a point mutation converting the conserved GGDEF motif to GADEF (*cdgA* ^G287A^) in cells also expressing GFP or GFP-VpsT. Expression of CdgA^G287A^ in *E. coli* was not able to recover VpsT localization, in contrast to wild type CdgA (Figure 4A, 4B and S9). Furthermore, the motility zone diameter of a strain expressing CdgA^G287A^ was similar to that of a strain with vector alone (Figure 4C). As expected, strains expressing GFP alone with the same compatible plasmids showed no localization pattern (Figure 4A, 4B and S9). These results suggest that the catalytic activity of CdgA as a DGC is required for VpsT localization.

**Figure 5:**
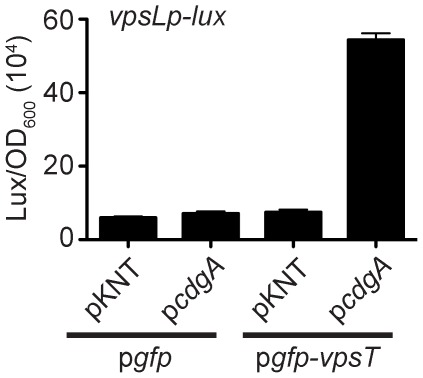
Co-expression of VpsT and CdgA Activates *vpsL* Expression in *E. coli.* Expression of the *vpsL* promoter fused to a *lux* reporter operon in *E. coli* expressing GFP or GFP-VpsT and harboring pKNT vector alone or pKNT containing *cdgA*. Expression is reported in luminescence counts min^−1^ ml^−1^/OD_600 nm_. One representative experiment of three biological replicates is shown. Error bars indicate standard deviations of four technical replicates.

We show above that the wild-type VpsT localization in *V. cholerae* is dependent on an intact DNA binding domain. To test whether VpsT requires DNA binding in *E. coli*, we expressed GFP-VpsT with a plasmid harboring the *vpsL* promoter (*vpsLp*). However, this strain did not exhibit a VpsT localization pattern (Figure 4A, 4B and S9). To determine whether VpsT requires both CdgA and a native DNA binding region, we expressed GFP-VpsT in cells containing a plasmid with both *cdgA* and the *vpsL* promoter. In this strain, GFP-VpsT appeared to form a more discrete pattern, exhibited an increased number of spots per cell, and a higher maximum to average intensity ratio when compared to GFP-VpsT cells co-expressing only CdgA (Figure 4A, 4B and S9). *E. coli* co-expressing GFP alone with the same compatible plasmids showed no localization pattern (Figure 4A, 4B and S9). To further determine whether GFP-VpsT activity requires c-di-GMP in *E. coli*, we quantified the expression of *vpsL* in the presence and absence of CdgA. Only *E. coli* co-expressing GFP-VpsT and CdgA activated *vpsL* expression while a strain expressing only GFP-VpsT did not show *vpsL* activation (Figure 5). These results suggest that VpsT localization is enhanced by DNA binding and requires elevated c-di-GMP levels to activate gene expression.

We then wondered whether CdgA, as a *V. cholerae* DGC, is required for VpsT localization or if a heterologous DGC could induce VpsT to localize. We therefore expressed *adrA*, a previously characterized gene encoding a DGC from *Salmonella typhimurium* [43], in strains also containing GFP or GFP-VpsT. Strains expressing AdrA showed a marked decrease in motility (Figure 4C), indicating that AdrA is functional in *E. coli*. In *E. coli*, AdrA caused GFP-VpsT to localize to foci, similar to foci induced by CdgA (Figure 4A, 4B and S9). As expected, co-expression of GFP and AdrA showed no localization. These results indicate that VpsT localization depends on the cellular level of c-di-GMP, and not on the presence of a specific *V. cholerae* DGC. Altogether, our results suggest that a direct interaction is not required for c-di-GMP signal transduction between DGCs and c-di-GMP receptors.

Recently, the subcellular localization of other c-di-GMP receptors was found to be dependent on c-di-GMP binding. C-di-GMP controls the subcellular localization of the PilZ domain containing c-di-GMP receptor YcgR in *E. coli*, where interaction of a YcgR-c-di-GMP complex with the flagellar motor leads to decreased motility and counter-clockwise rotational bias [8]–[10]. Moreover, multiple DGCs were shown to contribute additively to these motility phenotypes [8]. In *Caulobacter crescentus*, c-di-GMP binding to a conserved I-site of PopA mediates the sequestration of this protein to the cell pole, where PopA facilitates cell cycle progression [44]. No single deletion of a GGDEF or EAL domain containing protein was sufficient to alter PopA localization [44]. However, the combined activity of two DGCs, PleD and DgcB, was shown to alter cell cycle dynamics [45]. The subcellular localization of YcgR and PopA appears to be modulated by the additive activity of multiple DGCs in combination, similar to our findings with VpsT.

**Figure 6:**
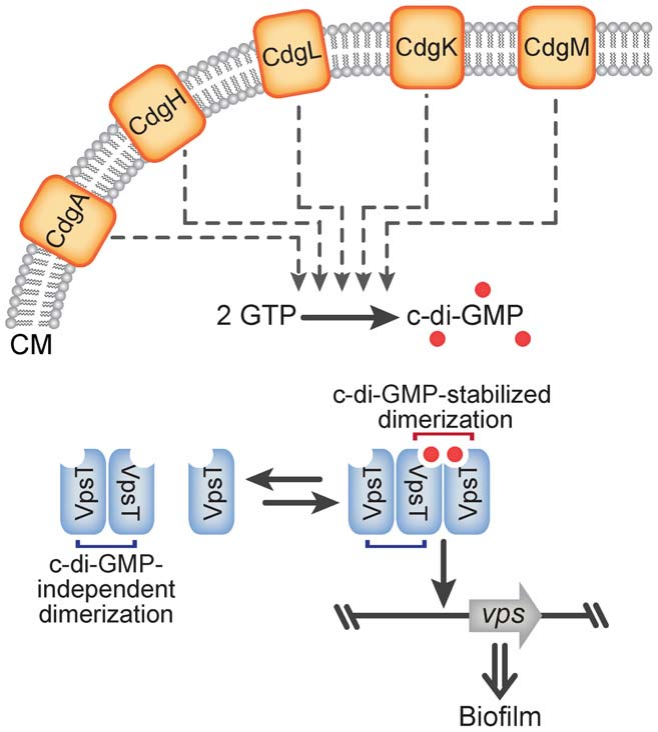
Model of c-di-GMP Signal Transduction in *V. cholerae*. Cellular c-di-GMP levels modulate VpsT oligomerization and subcellular localization. The additive effect of 5 membrane-bound DGCs regulates cellular c-di-GMP levels modulating VpsT oligomerization state, localization and DNA binding. Cytoplasmic membrane (CM).

This study is the first account of the subcellular localization of a c-di-GMP binding transcriptional regulator. Results presented here suggest that adequate levels of c-di-GMP contributed by multiple DGCs modulate VpsT activity and not a physical interaction or compartmentalization of c-di-GMP signaling components (Figure 6). This study identifies the requirements for signal transduction, localization and activity of a c-di-GMP receptor protein and furthers our understanding of the mechanisms of second messenger signaling.

## Materials and Methods

### Bacterial Strains, Plasmids and Culture Conditions

The bacterial strains and plasmids used in this study are listed in Table S1. In-frame deletion, chromosomal fusion and point mutation strains were generated according to previously published protocols [46]. All *V. cholerae* and *E. coli* strains were grown aerobically, at 30°C and 37°C, respectively, unless otherwise noted. Growth medium consisted of LB media (1% Tryptone, 0.5% Yeast Extract, 1% NaCl), pH 7.5. LB-agar and soft agar plates contained 1.5% and 0.3% (wt/vol) granulated agar (Difco), respectively. Concentrations of antibiotics used, where appropriate, were as follows: ampicillin (100 µg/ml), rifampicin (100 µg/ml), chloramphenicol (*E. coli* 20 µg/ml, *V. cholerae* 5 µg/ml), kanamycin (50 µg/ml) and gentamicin (30 µg/ml).

### Recombinant DNA Techniques

All strains were verified by PCR. Plasmid sequences were verified by DNA sequencing by Sequetech Corporation (Mountain View, CA). Primers used in the present study were purchased from Bioneer Corporation (Alameda, CA) and sequences are available upon request.

### Fluorescence Microscopy and Quantification

*V. cholerae* cells harboring the indicated plasmid were grown overnight (15 to 17 h) aerobically in LB medium supplemented with ampicillin. Cells were then diluted 1∶1000 in fresh LB medium and grown aerobically for 2 h, at which point arabinose was added at a final concentration of 0.05% and cells were harvested at exponential phase 2 h later (optical density at 600 nm (OD_600 nm_) of 0.2 to 0.4). *E. coli* cells containing the indicated plasmid were grown overnight in LB medium containing 2% glucose, kanamycin and ampicillin. Cells were then diluted 1∶50 in fresh LB medium containing 0.1% arabinose and 100 µM IPTG and cells were harvested 3 h later. Cell culture was spotted onto 1% agarose pads prepared with phosphate-buffered saline (PBS), pH 7.4. Images were acquired using a Zeiss Axiovert 200 microscope equipped with a 63× Plan-Apochromat objective (numerical aperture, 1.4), and were recorded with a Cool-Snap HQ2 camera (Photometrics). Images were minimally processed using Adobe Photoshop 11.0 and ImageJ_NIH_ software. MicrobeTracker [25] was employed, using the alg4ecoli parameter to identify cell outlines, the spotFinderZ tool to determine the number of spots per cell and the intprofile tool to determine the maximum and average fluorescence intensities of single cells. Data were acquired from at least 3 independent experiments and quantification was performed on at least 150 cells per treatment. All statistics were calculated using Graphpad Prism 4.

### Cellular Fractionation and Immunoblot

Overnight cultures were diluted 1∶200, grown to an OD_600 nm_ of 0.3 to 0.4, and diluted again 1∶200. Cells were harvested at an OD_600 nm_ of 0.3 to 0.4 by centrifugation (10,000× g) and fractionation was carried out as described previously [47]. Protein levels were quantified using a bicinchoninic acid (BCA) kit (Thermo Fisher Scientific Inc.) and normalized between fractions. Proteins were separated on a 12% SDS-polyacrylamide gel and electroblotted onto a nitrocellulose membrane with a Mini Trans-Blot Cell (Bio-Rad) as described previously [47]. Rabbit polyclonal antiserum against *V. cholerae* OmpU (provided by K. Klose) was used at a dilution of 1∶100,000. Mouse monoclonal antibody against GFP (Santa Cruz Biotechnology) and rabbit polyclonal antibody against the HA epitope (Santa Cruz Biotechnology) were used according to the manufacturer's instructions. Horseradish peroxidase-conjugated goat anti-rabbit secondary antibody (Santa Cruz Biotechnology) or goat anti-mouse secondary antibody (Santa Cruz Biotechnology) was used according to the manufacturer's instructions. Immunoblot analyses were conducted with at least three biological replicates.

### β-galactosidase Assays

β-galactosidase assays were performed and Miller units calculated as described previously [48]. The assays were repeated with three biological replicates and six technical replicates.

### Luminescence Assays

*V. cholerae* or *E. coli* cells harboring the indicated plasmid were grown overnight (15 to 17 h) aerobically in LB medium supplemented with the appropriate antibiotics. Cells were then diluted 1∶1000 in fresh LB medium and harvested at exponential phase at an OD_600 nm_ of 0.3 to 0.4. *E. coli* were grown in the presence of 0.1% arabinose and 100 µM IPTG for protein expression. Luminescence was measured using a Victor3 Multilabel Counter (PerkinElmer) and Lux expression is reported as counts min^−1^ ml^−1^/OD_600 nm_. Assays were repeated with at least three biological replicates and four technical replicates.

### Quantification of Cellular c-di-GMP Levels

Cellular c-di-GMP levels were measured in the indicated strains grown to exponential phase in LB medium. Protein concentration was determined using a BCA kit according to the manufacturer's instructions. C-di-GMP extraction, analysis by high-performance liquid chromatography-tandem mass spectrometry (HPLC-MS/MS) and c-di-GMP standard curve generation were carried out as described previously [26]. C-di-GMP quantification was performed with at least three biological replicates.

### Bacterial Two-hybrid Assay

Bacterial two-hybrid assays were performed as described previously [38]. Translational fusions were created with proteins of interest and T18 or T25 fragments of *B. pertussis* adenylate cyclase (CyaA). All constructs were confirmed by DNA sequencing. Plasmids pKT25-zip and pUT18C-zip, each containing translational fusions to the leucine zipper of GCN4, were used as positive controls. Production of cAMP by reconstituted CyaA was observed in the *E. coli* strain BTH101, lacking a native *cyaA* gene. Protein-protein interactions were observed by growing cells for 48 to 72 h at 30°C on LB agar containing ampicillin (100 µg/ml), kanamycin (50 µg/ml), X-gal (40 µg/ml) and IPTG (10 to 500 µM), or quantified by performing β-galactosidase assays with cells grown overnight at 30°C in LB medium containing ampicillin (100 µg/ml), kanamycin (50 µg/ml) and IPTG (10 µM).

### Accession Numbers

GenBank accession numbers are as follows: VpsT, NP_233336.1; VpsL, NP_230581.1; CdgA, NP_232475.1; CdgH, NP_230712.1; CdgK, NP_230749.1; CdgL, NP_231916.1; CdgM, NP_231020.1; MbaA, NP_230352.1; RocS, NP_230302.1; CdgC, NP_233171.1.

## Supporting Information

Figure S1 **Localization of GFP-VpsT in strains lackingc genes encoding DGCs important for *vps* expression.** Localization of GFP-VpsT in wild-type *V. cholerae* (Wt), strains with in-frame deletions of the genes encoding DGCs *cdgA*, *cdgH*, *cdgK*, *cdgL*, *cdgM* or a strain where all 5 DGCs are deleted in combination (Δ5DGC). Wild type expressing GFP is included as a fluorescence localization control. (A) Representative epifluorescence micrographs of the indicated strains are shown. Marker is 2 µm. (B) The number of spots per cell is shown as a histogram for the strains indicated. Data are acquired from at least 3 independent experiments and quantification was performed on at least 150 cells per treatment. (C) The ratio of maximum to average fluorescence intensity across the length of individual cells is shown as box plots for the indicated strains. Upper quartile, median and lower quartiles are indicated by top, middle and bottom lines of boxes, respectively, largest and smallest observations are indicated by lines above and below boxes, circles are outliers. Data are acquired from at least 3 independent experiments and quantification was performed on at least 150 cells per treatment. *, p<0.0001 using a student's t-test. (D) Protein levels of GFP-VpsT relative to GFP alone in wild type and Δ5DGC. Strains were grown in the same conditions as those used for fluorescent subcellular localization as described in the materials and methods. Equal amounts of protein from each sample were separated on a SDS-polyacrylamide gel, electroblotted onto a nitrocellulose membrane and detected using a monoclonal antibody against GFP (Santa Cruz Biotechnology) and an HRP-conjugated secondary antibody. Band intensities were quantified using ImageQuant software (Molecular Dynamics). Data indicate the average of at least three biological replicates and error bars indicate standard error.
(TIF)

Figure S2 **Census of** ***vps*** **expression in strains with deletions in each gene encoding a GGDEF and/or EAL domain containing protein in *V. cholerae.*** Expression of *vpsL* in 52 strains containing in-frame deletions of each gene in *V. cholerae* genome encoding proteins containing GGDEF (A), EAL (B) or GGDEF and EAL (C) domains. Expression of *vpsL* was quantified using a *vpsLp-lux* operon transcriptional fusion on a plasmid in wild-type *V. cholerae* (Wt) or strains containing in-frame deletions of each gene indicated. Cells were grown to exponential phase (OD_600 nm_ of 0.3 to 0.4) in LB media containing chloramphenicol (5 µg/ml). Expression is reported in luminescence counts min^−1^ ml^−1^/OD_600 nm_. Error bars indicate standard deviations of four technical replicates. One representative experiment is shown of at least three biological replicates.
(TIF)

Figure S3 **A single DGC can rescue** ***vpsL*** **expression in the Δ5DGC strain.** Expression of a chromosomal *vpsL* promoter-*lacZ* fusion was measured in wild type (Wt) or Δ5DGC *V. cholerae* strains containing pBAD vector alone, or pBAD containing *cdgA* using β-galactosidase assays. One representative experiment of three biological replicates is shown. Error bars indicate standard deviations of eight technical replicates.
(TIF)

Figure S4 **Localization of GFP-VpsT in strains lacking genes encoding PDEs important for** ***vps*** **expression.** Localization of GFP-VpsT in wild-type *V. cholerae* (Wt), in-frame deletion strains of the genes encoding PDEs *cdgC*, *mbaA*, *rocS*, or a strain where all 3 PDEs are deleted in combination (Δ3PDE). Wild type expressing GFP is included as a fluorescence localization control. (A) Representative epifluorescence micrographs are shown. Marker is 2 µm. (B) The number of spots per cell is shown as a histogram for the strains indicated. Data are acquired from at least 3 independent experiments and quantification was performed on at least 150 cells per treatment. (C) The ratio of maximum to average fluorescence intensity across the length of individual cells is shown as box plots for the indicated strains. Data are acquired from at least 3 independent experiments and quantification was performed on at least 150 cells per treatment.
(TIF)

Figure S5 **Complementation with GFP-fusion or HA-epitope tagged proteins.** Relative expression, compared to wild type (Wt), of a chromosomal *vpsL* promoter fusion to *lacZ* in (A) wild type carrying pBAD vector or Δ*cdgA* strains carrying pBAD vector or pBAD containing a *cdgA-gfp* fusion, or (B) wild type carrying pBAD vector or Δ*cdgH* strains carrying pBAD vector or pBAD containing a *cdgH-gfp* fusion. Cells were grown to exponential phase (OD_600 nm_ of 0.3 to 0.4) in LB broth containing ampicillin (100 µg/ml) and arabinose (0.01 to 0.05%). Error bars indicate standard deviation of at least 6 technical replicates. The results shown are one representative experiment of three biological replicates. Expression of a *vpsL* promoter fusion to a *lux* operon in (C) wild type, Δ*vpsT* or chromosomal HA-*vpsT* strains, (D) wild type, Δ*cdgA* or chromosomal *cdgA*-HA strains or (E) wild type, Δ*cdgH* or chromosomal *cdgH*-HA strains. Error bars indicate standard deviation of at least 4 technical replicates. The results shown are one representative experiment of three biological replicates.
(TIF)

Figure S6 **The cellular localization of VpsT is not altered in the Δ5DGC Strain.** Subcellular fractionation of *V. cholerae* wild type (Wt) or Δ5DGC strains containing *vpsT* tagged with an HA epitope in the native *vpsT* locus. Western immunoblot was performed on cellular fractions representing whole cell (WC), cytoplasmic (C) and total membrane (M) fractions. HA-VpsT was detected using a polyclonal anti-HA antibody. *gfp* was constitutively expressed from a chromosomal locus. GFP was detected using monoclonal anti-GFP antibody and is used as a cytoplasmic fraction control. OmpU was detected using a polyclonal anti-OmpU antibody and is used as a total membrane fraction control. One representative experiment of three biological replicates is shown.
(TIF)

Figure S7 **VpsT does not interact directly with CdgA or CdgH.** *vpsT* was cloned into vectors pUT18C or pKT25 creating plasmids expressing full length VpsT, tagged at its N-terminus with T18 or T25 fragments of *B. pertussis* adenylate cyclase (*cyaA*). *cdgA* or *cdgH* was cloned into vectors pUT18 or pKNT25, creating proteins tagged at their C-termini with T18 or T25. Empty vectors or those containing fusion proteins were co-transformed into *E. coli* strain BTH101. Quantification of bacterial two-hybrid interactions was performed by β-galactosidase assays on cells containing the indicated plasmids grown overnight at 30°C in LB broth containing ampicillin (100 µg/ml), kanamycin (50 µg/ml) and IPTG (10 µM). pKT25-zip and pUT18C-zip contain genes encoding the GCN4 leucine zipper as a positive protein-protein interaction control.
(TIF)

Figure S8 **Localization of GFP-VpsT point mutants.** (A) Single-cell quantification of GFP-VpsT subcellular localization. The ratio of maximum to average fluorescence intensity across the length of individual cells is shown as box plots for Δ*vpsT* strains expressing GFP, GFP-VpsT or GFP-VpsT containing the indicated point mutations. Data are acquired from at least 3 independent experiments and quantification was performed on at least 150 cells per treatment. *, p<0.0001 using a student's t-test. (B) Protein levels of wild type and mutant GFP-VpsT fusion proteins relative to GFP alone. Strains were grown in the same conditions as those used for fluorescent subcellular localization as described in the materials and methods. Equal amounts of protein from each sample were separated on a SDS-polyacrylamide gel, electroblotted onto a nitrocellulose membrane and detected using a monoclonal antibody against GFP (Santa Cruz Biotechnology) and an HRP-conjugated secondary antibody. Band intensities were quantified using ImageQuant software (Molecular Dynamics). Data indicate the average of at least three biological replicates and error bars indicate standard error. (C) VpsT helix-turn-helix sequence alignment. Sequence alignment, using ClustalW, of the VpsT helix-turn-helix region with other members of the LuxR/FixJ superfamily of transcription factors. Protein sequences used to generate the alignment are as follows: *Vibrio cholerae* O1 El Tor N16961 VpsT (NP_233336), *Vibrio fischeri* MJ11 LuxR (YP_002158591), *Escherichia coli* O157:H7 NarL (NP_287469), *Sinorhizobium meliloti* 1021 FixJ (NP_435915), *Bacillus subtilis* subsp. *subtilis* str. 168 DegU (NP_391429). Numbers to the left of each protein name correspond to the starting amino acid number of each protein. Arrow indicates residue H193 of VpsT.
(TIF)

Figure S9 **Subcellular localization of VpsT expressed in** ***E. coli*** **.** The ratio of maximum to average fluorescence intensity across the length of individual cells is shown as box plots for strains expressing GFP (open box) or GFP-VpsT (shaded box) and containing pKNT25 or pKNT25 harboring *cdgA*, *cdgA* ^G287A^, the *vpsL* promoter (*vpsLp*), *cdgA* and *vpsLp* or *adrA*. Data are acquired from at least 3 independent experiments and quantification was performed on at least 150 cells per treatment. *, p<0.0001 using a student's t-test.
(TIF)
