# Correction: Cellular Levels and Binding of c-di-GMP Control Subcellular Localization and Activity of the *Vibrio cholerae* Transcriptional Regulator VpsT

**DOI:** 10.1371/annotation/6d6bf70a-03e1-4612-b75e-b89bd334fbc4

**Published:** 2012-07-03

**Authors:** Nicholas J. Shikuma, Jiunn C. N. Fong, Fitnat H. Yildiz

Supporting Table S1, Bacterial Strains and Plasmids, does not appear. Please view Table S1 here: 

The legend for Figure 9 is: Bacterial strains and plasmids used in this work. 

