# Supplementary figures and images for: Cellular Levels and Binding of c-di-GMP Control Subcellular Localization and Activity of the Vibrio cholerae Transcriptional Regulator VpsT
Source: PLoS Pathog. 2012 May 24;8(5):e1002719. doi: 10.1371/journal.ppat.1002719 (PMC3359988; doi:10.1371/journal.ppat.1002719)

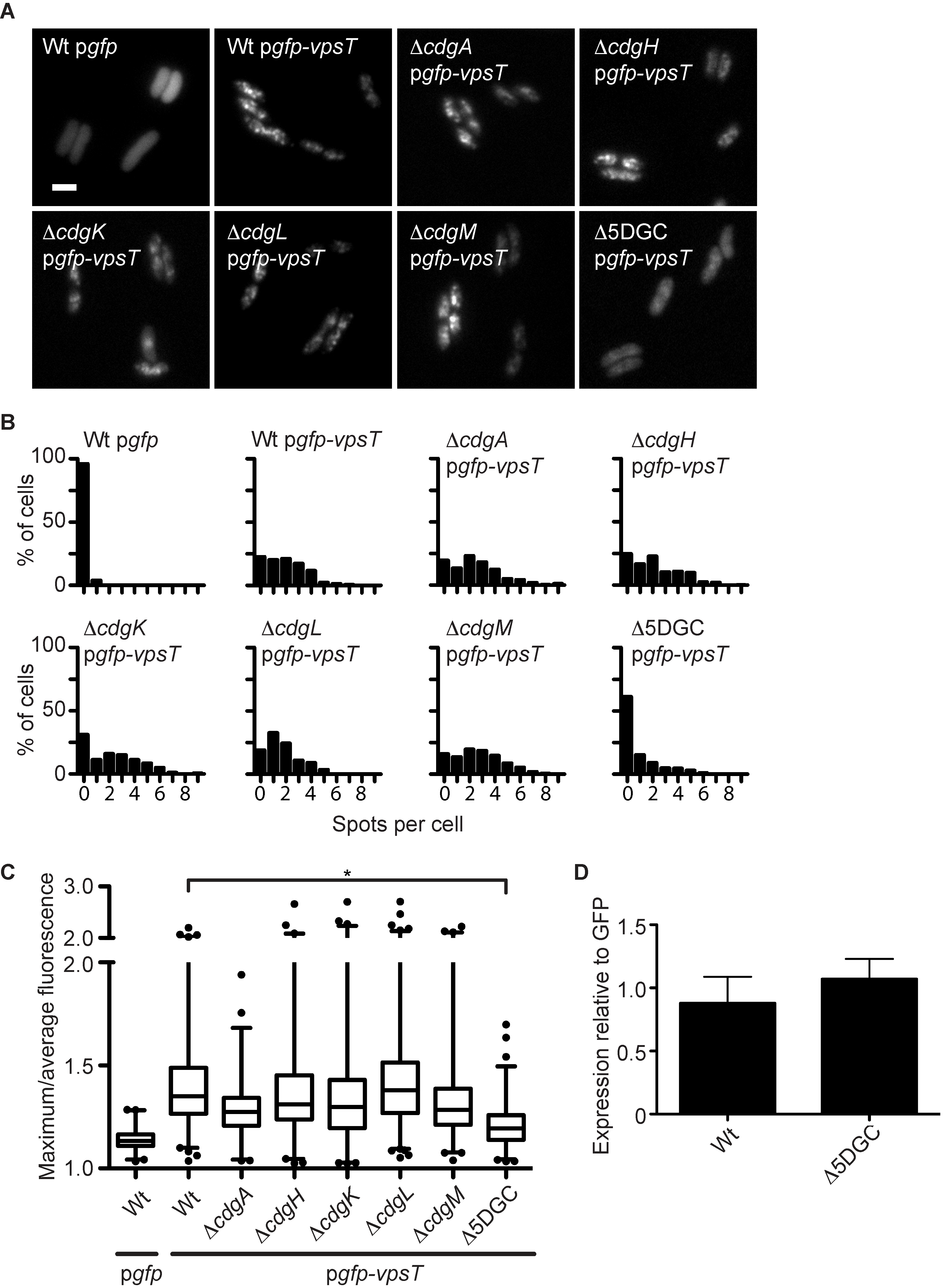

Supplement: Figure S1 — Localization of GFP-VpsT in strains lackingc genes encoding DGCs important for vps expression. Localization of GFP-VpsT in wild-type V. cholerae (Wt), strains with in-frame deletions of the genes encoding DGCs cdgA, cdgH, cdgK, cdgL, cdgM or a strain where all 5 DGCs are deleted in combination (Δ5DGC). Wild type expressing GFP is included as a fluorescence localization control. (A) Representative epifluorescence micrographs of the indicated strains are shown. Marker is 2 µm. (B) The number of spots per cell is shown as a histogram for the strains indicated. Data are acquired from at least 3 independent experiments and quantification was performed on at least 150 cells per treatment. (C) The ratio of maximum to average fluorescence intensity across the length of individual cells is shown as box plots for the indicated strains. Upper quartile, median and lower quartiles are indicated by top, middle and bottom lines of boxes, respectively, largest and smallest observations are indicated by lines above and below boxes, circles are outliers. Data are acquired from at least 3 independent experiments and quantification was performed on at least 150 cells per treatment. *, p<0.0001 using a student's t-test. (D) Protein levels of GFP-VpsT relative to GFP alone in wild type and Δ5DGC. Strains were grown in the same conditions as those used for fluorescent subcellular localization as described in the materials and methods. Equal amounts of protein from each sample were separated on a SDS-polyacrylamide gel, electroblotted onto a nitrocellulose membrane and detected using a monoclonal antibody against GFP (Santa Cruz Biotechnology) and an HRP-conjugated secondary antibody. Band intensities were quantified using ImageQuant software (Molecular Dynamics). Data indicate the average of at least three biological replicates and error bars indicate standard error. (TIF) [file ppat.1002719.s001.tif]

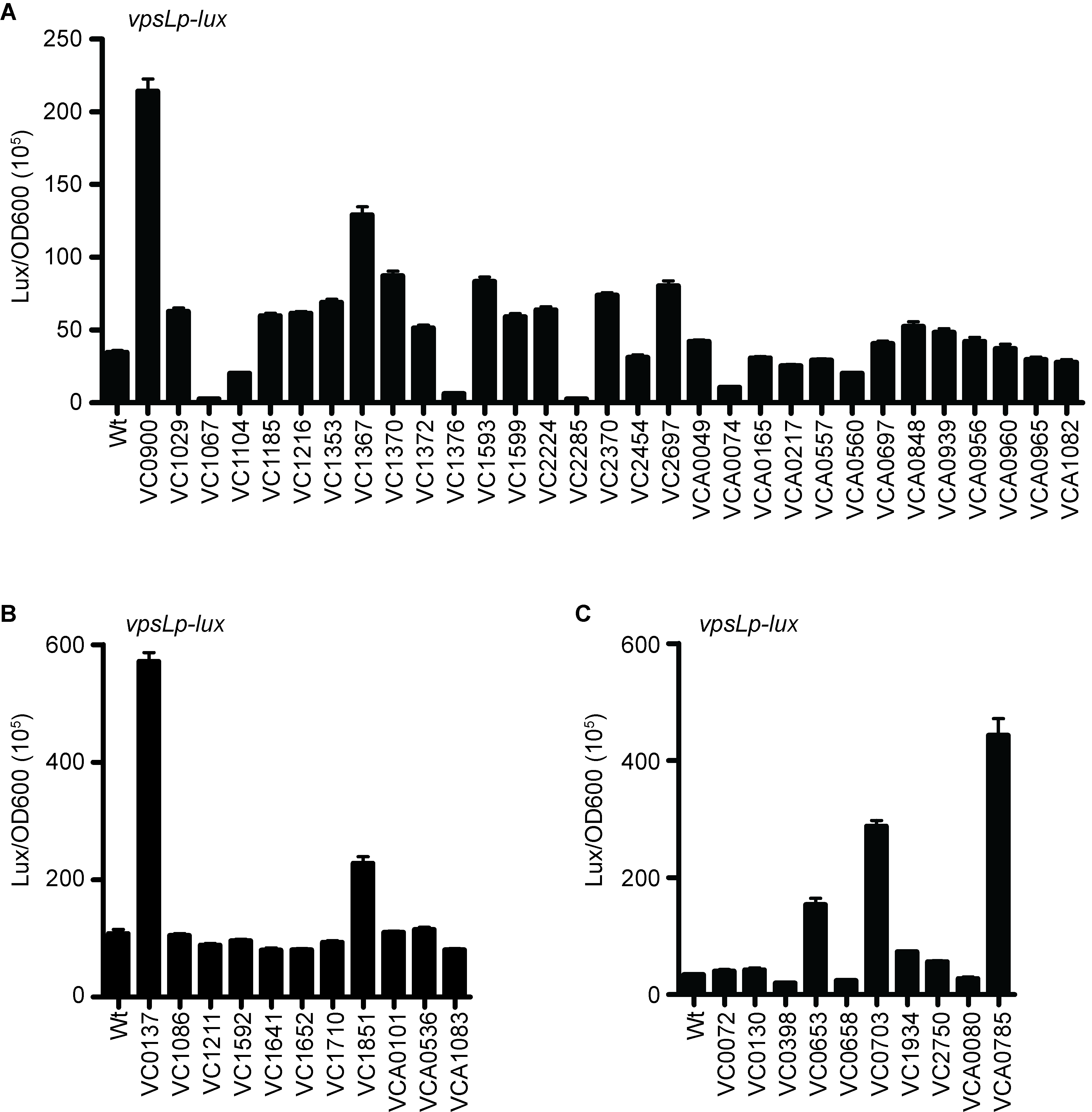

Supplement: Figure S2 — Census of vps expression in strains with deletions in each gene encoding a GGDEF and/or EAL domain containing protein in V. cholerae. Expression of vpsL in 52 strains containing in-frame deletions of each gene in V. cholerae genome encoding proteins containing GGDEF (A), EAL (B) or GGDEF and EAL (C) domains. Expression of vpsL was quantified using a vpsLp-lux operon transcriptional fusion on a plasmid in wild-type V. cholerae (Wt) or strains containing in-frame deletions of each gene indicated. Cells were grown to exponential phase (OD600 nm of 0.3 to 0.4) in LB media containing chloramphenicol (5 µg/ml). Expression is reported in luminescence counts min−1 ml−1/OD600 nm. Error bars indicate standard deviations of four technical replicates. One representative experiment is shown of at least three biological replicates. (TIF) [file ppat.1002719.s002.tif]

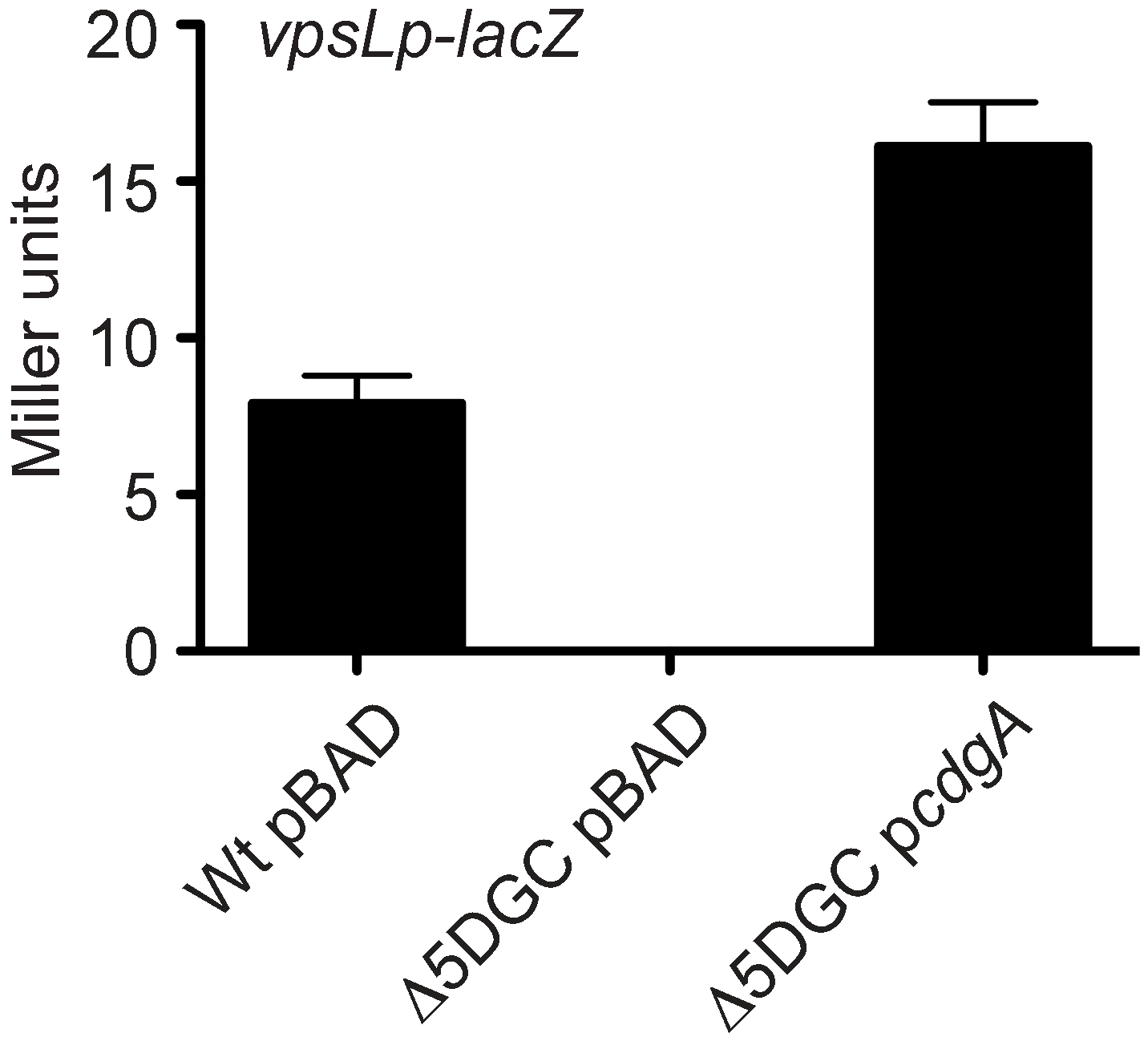

Supplement: Figure S3 — A single DGC can rescue vpsL expression in the Δ5DGC strain. Expression of a chromosomal vpsL promoter-lacZ fusion was measured in wild type (Wt) or Δ5DGC V. cholerae strains containing pBAD vector alone, or pBAD containing cdgA using β-galactosidase assays. One representative experiment of three biological replicates is shown. Error bars indicate standard deviations of eight technical replicates. (TIF) [file ppat.1002719.s003.tif]

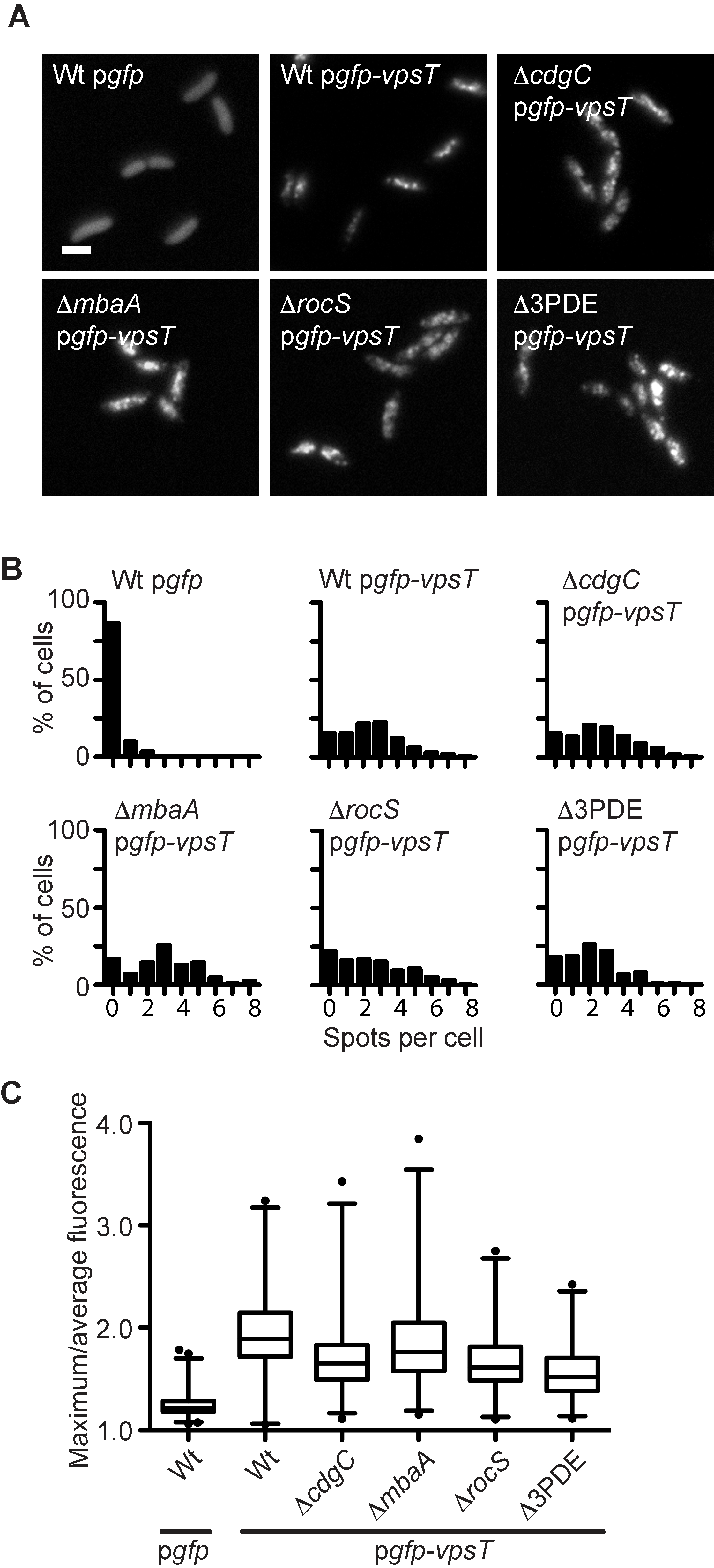

Supplement: Figure S4 — Localization of GFP-VpsT in strains lacking genes encoding PDEs important for vps expression. Localization of GFP-VpsT in wild-type V. cholerae (Wt), in-frame deletion strains of the genes encoding PDEs cdgC, mbaA, rocS, or a strain where all 3 PDEs are deleted in combination (Δ3PDE). Wild type expressing GFP is included as a fluorescence localization control. (A) Representative epifluorescence micrographs are shown. Marker is 2 µm. (B) The number of spots per cell is shown as a histogram for the strains indicated. Data are acquired from at least 3 independent experiments and quantification was performed on at least 150 cells per treatment. (C) The ratio of maximum to average fluorescence intensity across the length of individual cells is shown as box plots for the indicated strains. Data are acquired from at least 3 independent experiments and quantification was performed on at least 150 cells per treatment. (TIF) [file ppat.1002719.s004.tif]

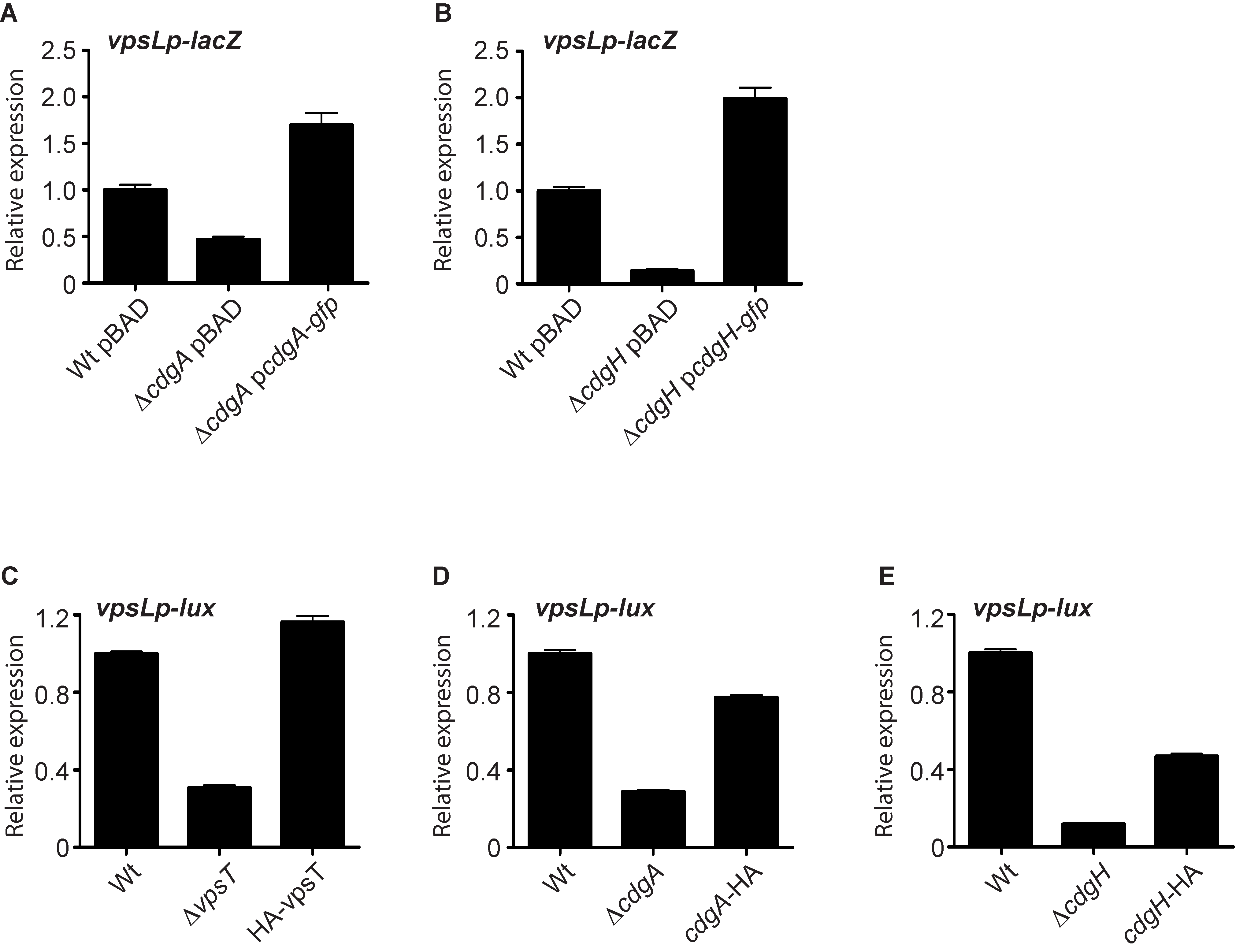

Supplement: Figure S5 — Complementation with GFP-fusion or HA-epitope tagged proteins. Relative expression, compared to wild type (Wt), of a chromosomal vpsL promoter fusion to lacZ in (A) wild type carrying pBAD vector or ΔcdgA strains carrying pBAD vector or pBAD containing a cdgA-gfp fusion, or (B) wild type carrying pBAD vector or ΔcdgH strains carrying pBAD vector or pBAD containing a cdgH-gfp fusion. Cells were grown to exponential phase (OD600 nm of 0.3 to 0.4) in LB broth containing ampicillin (100 µg/ml) and arabinose (0.01 to 0.05%). Error bars indicate standard deviation of at least 6 technical replicates. The results shown are one representative experiment of three biological replicates. Expression of a vpsL promoter fusion to a lux operon in (C) wild type, ΔvpsT or chromosomal HA-vpsT strains, (D) wild type, ΔcdgA or chromosomal cdgA-HA strains or (E) wild type, ΔcdgH or chromosomal cdgH-HA strains. Error bars indicate standard deviation of at least 4 technical replicates. The results shown are one representative experiment of three biological replicates. (TIF) [file ppat.1002719.s005.tif]

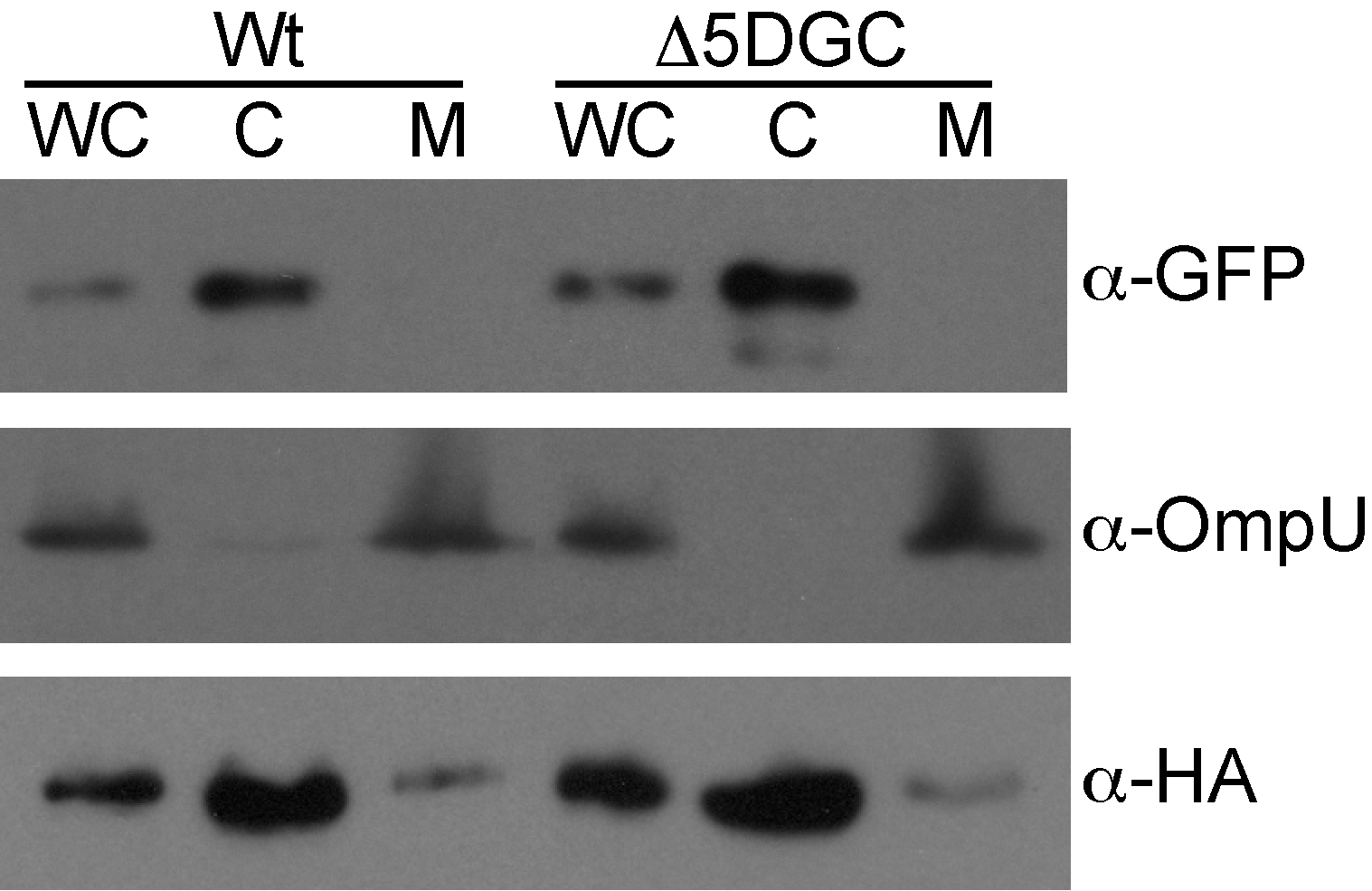

Supplement: Figure S6 — The cellular localization of VpsT is not altered in the Δ5DGC Strain. Subcellular fractionation of V. cholerae wild type (Wt) or Δ5DGC strains containing vpsT tagged with an HA epitope in the native vpsT locus. Western immunoblot was performed on cellular fractions representing whole cell (WC), cytoplasmic (C) and total membrane (M) fractions. HA-VpsT was detected using a polyclonal anti-HA antibody. gfp was constitutively expressed from a chromosomal locus. GFP was detected using monoclonal anti-GFP antibody and is used as a cytoplasmic fraction control. OmpU was detected using a polyclonal anti-OmpU antibody and is used as a total membrane fraction control. One representative experiment of three biological replicates is shown. (TIF) [file ppat.1002719.s006.tif]

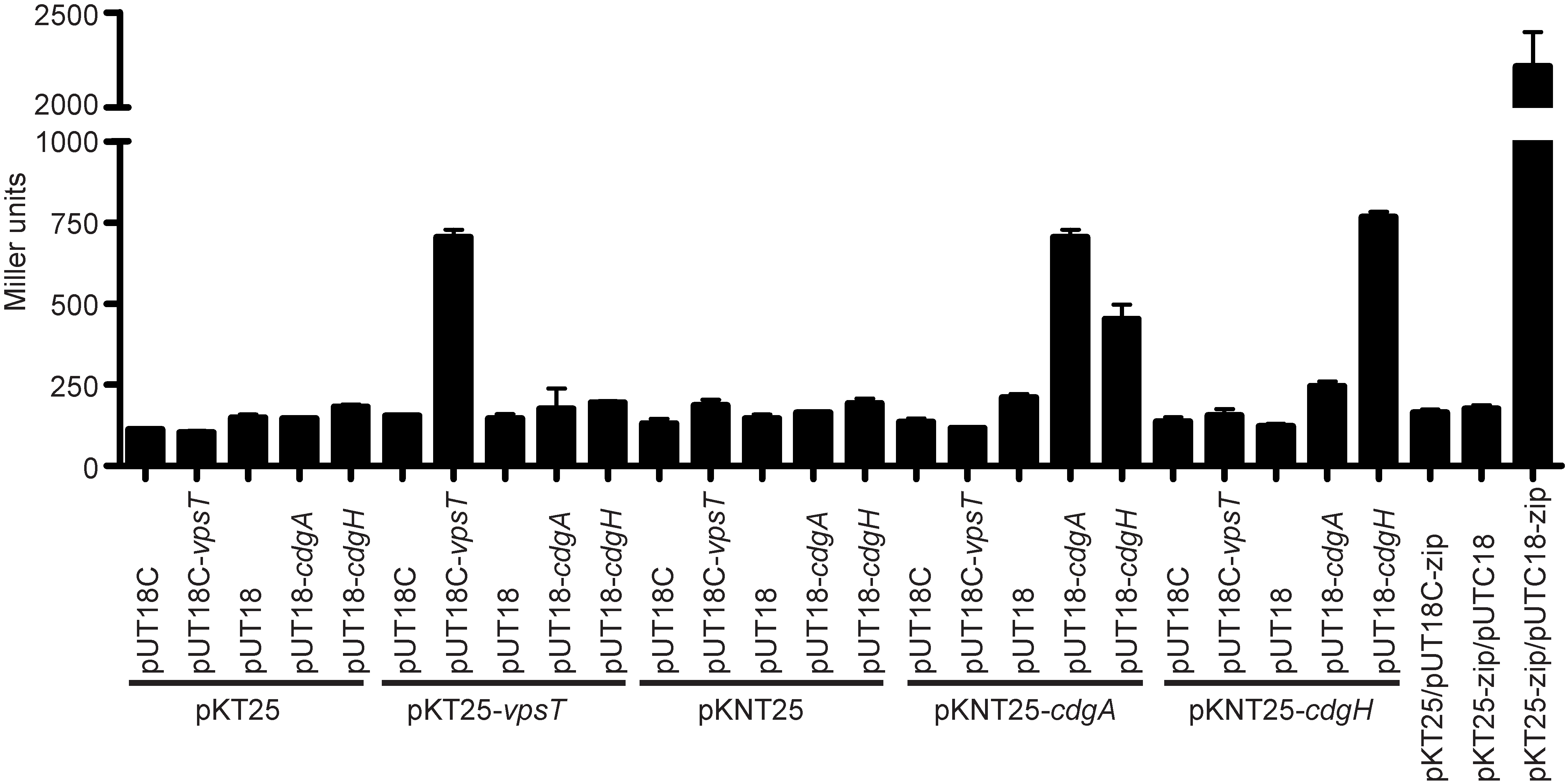

Supplement: Figure S7 — VpsT does not interact directly with CdgA or CdgH. vpsT was cloned into vectors pUT18C or pKT25 creating plasmids expressing full length VpsT, tagged at its N-terminus with T18 or T25 fragments of B. pertussis adenylate cyclase (cyaA). cdgA or cdgH was cloned into vectors pUT18 or pKNT25, creating proteins tagged at their C-termini with T18 or T25. Empty vectors or those containing fusion proteins were co-transformed into E. coli strain BTH101. Quantification of bacterial two-hybrid interactions was performed by β-galactosidase assays on cells containing the indicated plasmids grown overnight at 30°C in LB broth containing ampicillin (100 µg/ml), kanamycin (50 µg/ml) and IPTG (10 µM). pKT25-zip and pUT18C-zip contain genes encoding the GCN4 leucine zipper as a positive protein-protein interaction control. (TIF) [file ppat.1002719.s007.tif]

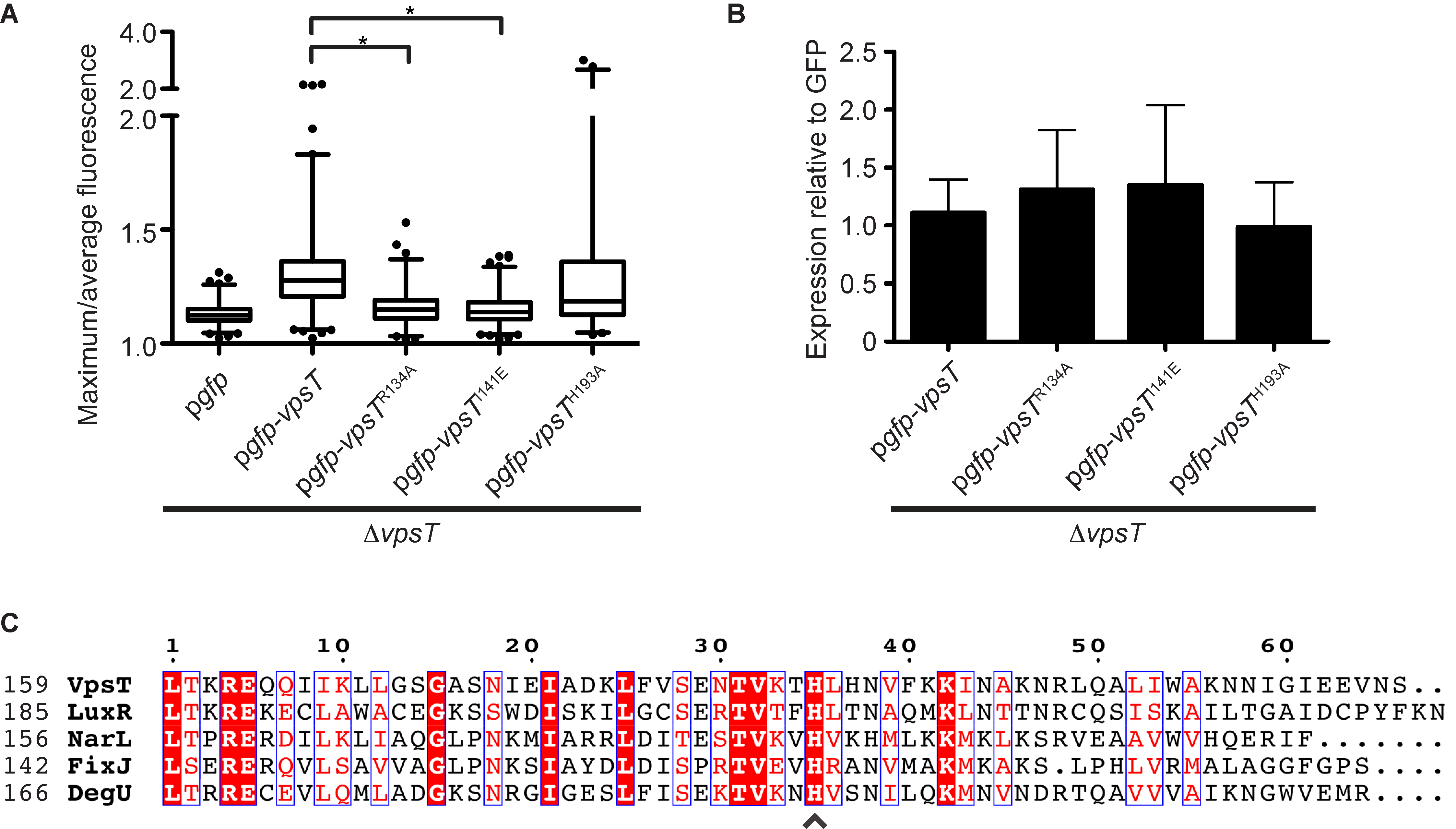

Supplement: Figure S8 — Localization of GFP-VpsT point mutants. (A) Single-cell quantification of GFP-VpsT subcellular localization. The ratio of maximum to average fluorescence intensity across the length of individual cells is shown as box plots for ΔvpsT strains expressing GFP, GFP-VpsT or GFP-VpsT containing the indicated point mutations. Data are acquired from at least 3 independent experiments and quantification was performed on at least 150 cells per treatment. *, p<0.0001 using a student's t-test. (B) Protein levels of wild type and mutant GFP-VpsT fusion proteins relative to GFP alone. Strains were grown in the same conditions as those used for fluorescent subcellular localization as described in the materials and methods. Equal amounts of protein from each sample were separated on a SDS-polyacrylamide gel, electroblotted onto a nitrocellulose membrane and detected using a monoclonal antibody against GFP (Santa Cruz Biotechnology) and an HRP-conjugated secondary antibody. Band intensities were quantified using ImageQuant software (Molecular Dynamics). Data indicate the average of at least three biological replicates and error bars indicate standard error. (C) VpsT helix-turn-helix sequence alignment. Sequence alignment, using ClustalW, of the VpsT helix-turn-helix region with other members of the LuxR/FixJ superfamily of transcription factors. Protein sequences used to generate the alignment are as follows: Vibrio cholerae O1 El Tor N16961 VpsT (NP_233336), Vibrio fischeri MJ11 LuxR (YP_002158591), Escherichia coli O157:H7 NarL (NP_287469), Sinorhizobium meliloti 1021 FixJ (NP_435915), Bacillus subtilis subsp. subtilis str. 168 DegU (NP_391429). Numbers to the left of each protein name correspond to the starting amino acid number of each protein. Arrow indicates residue H193 of VpsT. (TIF) [file ppat.1002719.s008.tif]

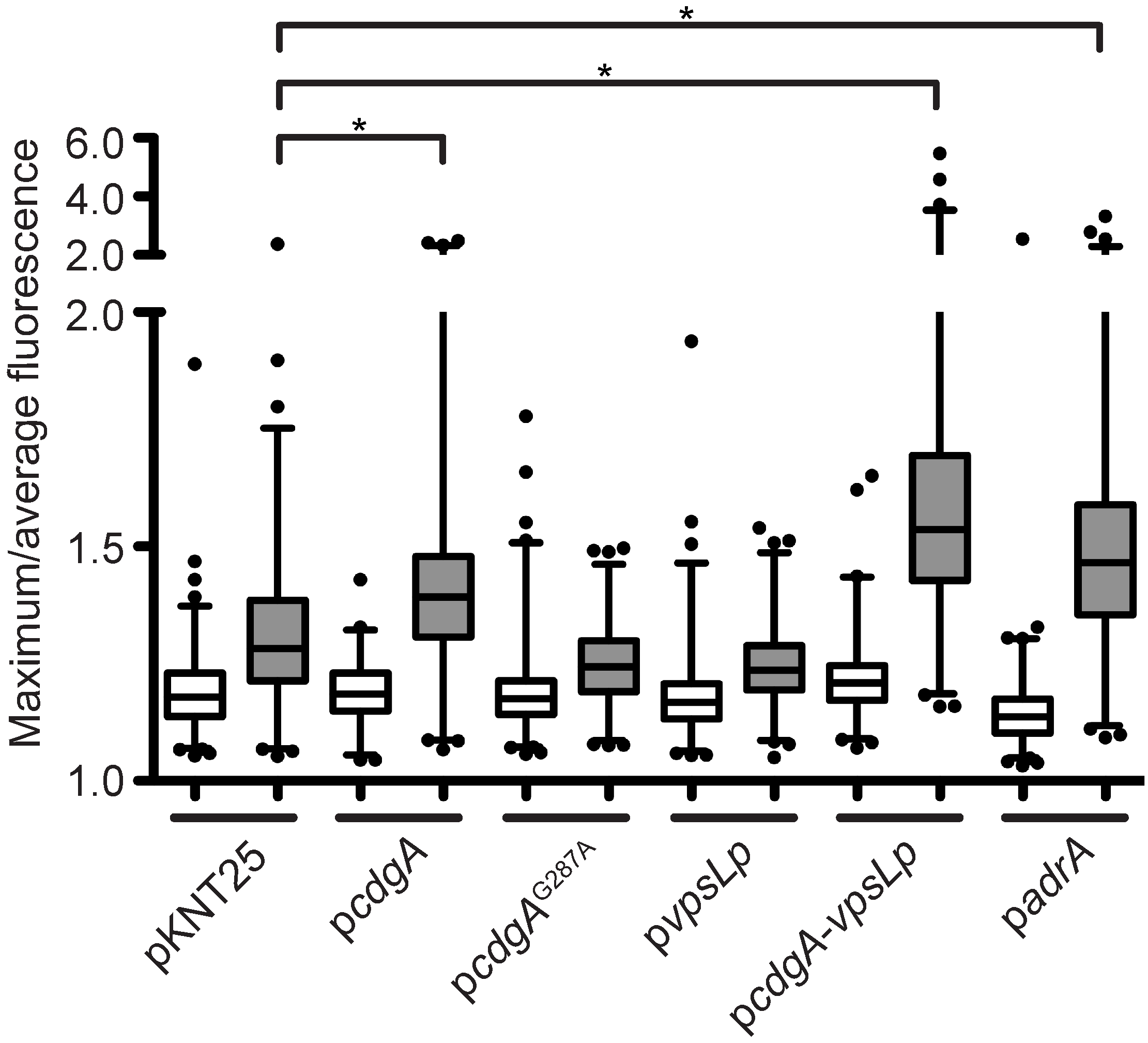

Supplement: Figure S9 — Subcellular localization of VpsT expressed in E. coli . The ratio of maximum to average fluorescence intensity across the length of individual cells is shown as box plots for strains expressing GFP (open box) or GFP-VpsT (shaded box) and containing pKNT25 or pKNT25 harboring cdgA, cdgA G287A, the vpsL promoter (vpsLp), cdgA and vpsLp or adrA. Data are acquired from at least 3 independent experiments and quantification was performed on at least 150 cells per treatment. *, p<0.0001 using a student's t-test. (TIF) [file ppat.1002719.s009.tif]
