# Supplementary figures and images for: Correction: Cellular Levels and Binding of c-di-GMP Control Subcellular Localization and Activity of the Vibrio cholerae Transcriptional Regulator VpsT
Source: PLoS Pathog. 2012 Jul 3;8(7):10.1371/annotation/6d6bf70a-03e1-4612-b75e-b89bd334fbc4. doi: 10.1371/annotation/6d6bf70a-03e1-4612-b75e-b89bd334fbc4 (PMC3392283; doi:10.1371/annotation/6d6bf70a-03e1-4612-b75e-b89bd334fbc4)

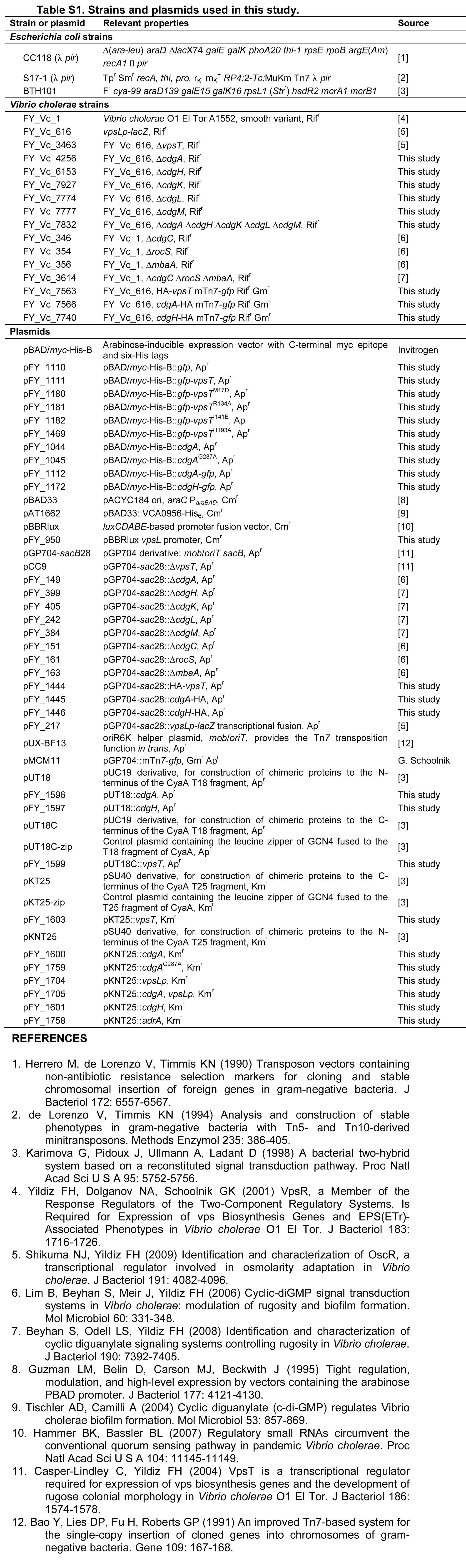

Supplement: Supplementary file 1 [file ppat.6d6bf70a-03e1-4612-b75e-b89bd334fbc4.s001.tif]
